# Vitamin D Deficiency and Associated Risk Factors in Women from Riyadh, Saudi Arabia

**DOI:** 10.1038/s41598-019-56830-z

**Published:** 2019-12-30

**Authors:** Nora A. AlFaris, Nora M. AlKehayez, Fatema I. AlMushawah, AbdulRhman N. AlNaeem, Nadia D. AlAmri, Ebtisam S. AlMudawah

**Affiliations:** 1 Nutrition and Food Science (PhD), Department of Physical Sport Science, Princess Nourah bint Abdulrahman University, Riyadh, P.O. Box 84428, Riyadh, 11671 Saudi Arabia; 2 King Fahad Medical City, P.O. Box 59046, Riyadh, 11525 Saudi Arabia; 3 King Saud Medical City, P.O. Box 3897, Riyadh, 11196 Saudi Arabia

**Keywords:** Malnutrition, Risk factors

## Abstract

Vitamin D deficiency is an epidemic public health problem worldwide. It is common in the Middle East and is more severe in women. This cross-sectional study was conducted to assess vitamin D deficiency and associated risk factors in women living in Riyadh, Saudi Arabia. Serum 25-hydroxyvitamin D (25(OH)D) was measured in 166 women aged 30–65 years. Socio-demographic, lifestyle and health status characteristics, as well as intake of selected dietary supplements, were collected. Weight and height were measured. Vitamin D deficiency (25(OH)D < 20 ng/mL) was reported in 60.2% of participants. Mean of serum 25(OH)D was 20.7 ng/mL. Older age and taking the supplements of vitamin D, multi-vitamins or calcium were identified as factors that associated with a lower risk of hypovitaminosis D. A national strategy is needed to control a hypovitaminosis D crisis in Saudi Arabia. This could be accomplished by raising public awareness regarding vitamin D, regulating and enhancing vitamin D fortification and supplementation and screening vitamin D status among women at high risk.

## Introduction

Vitamin D has a crucial role in calcium homeostasis and metabolism in the human body and consequently is considered important to maintain bone health^1^. Furthermore, vitamin D plays an essential role in the modulation of the immune system and the regulation of body cells’ differentiation and proliferation. Therefore, vitamin D deficiency is believed to be associated with the risk of several serious non-skeletal chronic diseases such as autoimmune diseases, cardiovascular disease and certain cancers^2^. Unfortunately, dietary sources of vitamin D are rare and found mainly in few foods such as fatty fish and fortified dairy products^3^. However, vitamin D can be obtained through synthesis in the human skin when the human body is exposed directly to ultraviolet B radiation of sunlight^3^. Vitamin D is produced in the human skin through photochemical conversion of 7-dehydrocholesterol to cholecalciferol (vitamin D3)^3^. Vitamin D3 is then metabolized to 25-hydroxyvitamin D (25(OH)D), the main storage and circulating form of the vitamin, and then to 1, 25-dihydroxyvitamin D, the hormonal form of the vitamin, by the hepatic and the renal enzymes^4^. In addition, there are alternative pathways of vitamin D activation by CYP11A1^5–8^. However, these products were not measured in this study.

Currently, the global prevalence of vitamin D deficiency is an epidemic and considered as a public health concern in many regions around the world^9^. The Middle East is a sunny region, but still suffering from a high prevalence of hypovitaminosis D^10^. In Saudi Arabia, widespread prevalence of vitamin D deficiency were reported in the different age and gender groups of this population, despite the plentiful sunshine that available throughout the year in this Middle Eastern country^11^. Furthermore, vitamin D deficiency is more prominent in women of varying ages in Saudi Arabia^12,13^. Several factors could contribute to vitamin D deficiency. Therefore, it is important to determine the risk factors that are associated with vitamin D deficiency among those women in order to establish relevant strategies to prevent and manage this serious health problem.

The objective of this study was to assess vitamin D deficiency among a sample of women living in Riyadh, Saudi Arabia and identified the major risk factors which might be associated with vitamin D deficiency by evaluating selected variables related to socio-demographic, lifestyle and health status characteristics, as well as, dietary supplements intake.

## Methods

### Study design and subjects

This study is a cross-sectional study. One hundred and sixty-eight women were recruited to participate from the King Saud Medical City in Riyadh, Saudi Arabia during the period from May 2015 to June 2016. The inclusion criteria were: women aged 30–65 years, living in Riyadh; Saudi Arabia (latitude 24.7°N) who had not been diagnosed with any medical disorder or taking any medications that interfere with vitamin D status. The systematic random sampling method was used to recruit the study sample from women who came for a follow-up examination at the gynecology clinic at the King Saud Medical City. In fact, two subjects were excluded as they didn’t meet the inclusion criteria, and therefore 166 women participated in the current study.

### Data collection

Data were collected from the participants directly by trained nutritionists. Collected data include socio-demographic characteristics such as age groups, lifestyle characteristics such as sun exposure duration, health status characteristics such as obesity, selected dietary supplements intake, as well as, measuring height and weight. The sun exposure frequency was defined as frequent when subjects were exposed to sunlight at least three times weekly, occasional when they were exposed to sunlight at least once per week, rare when they were exposed to sunlight at least once a month, and never when their sun exposure was lower than once per month. Sun exposure meant that subjects had to expose at least 20% of their bodies including the face, arms and hands to sunshine directly. Body height was measured to the closest 0.1 cm and body weight was measured to the closest 0.1 kg using standardized methods. Body mass index (BMI) was calculated by dividing weight (in kg) over height squared (in meter). Obesity was defined as a BMI equal to or higher than 30. Data regarding diagnoses of type 2 diabetes and/or hypertension were collected from the medical files of the patients.

### Biochemical analysis

After a 12- hour of overnight fasting, blood samples were collected from all participants at the midday at the clinical laboratory. Plasma was separated by centrifugation at 2000 g for 20 minutes within an hour after the samples were drawn and stored at −70 °C for further analysis. Vitamin D levels, 25(OH)D were determined by a radioimmunoassay technique (Roche Diagnostics, Mannheim, Germany). Subjects were classified based on vitamin D level into vitamin D sufficient (25(OH)D ≥ 30 ng/mL), insufficient (25(OH)D = 20–29.9 ng/mL), and deficient (25(OH)D < 20 ng/mL). Moreover, severe vitamin D deficiency was defined as 25(OH)D < 10 ng/mL^14^.

### Statistical analysis

SPSS version 20 was used for data analysis. Categorical variables were expressed as frequencies and percentages. Continuous variables were expressed as both mean ± standard deviation (SD) and median ± interquartile range (IQR). Univariate logistic regression analysis was conducted to identify risk factors which might be associated with vitamin D deficiency. Differences were considered statistically significant at *p* values < 0.05.

### Ethics approval and consent to participate

All participants were given informed consent in their native language to sign prior to enrolling in this study. All human protocols were approved by the King Saud Medical City Institutional Review Board in accordance with the declaration of Helsinki.

## Results

Overall, 166 women living in Riyadh; Saudi Arabia were involved in this study. The socio-demographic and lifestyle characteristics of the study subjects are presented in Table 1. Most of the study subjects (85.5%) are Saudis. Younger women aged 30–49 years formed 57.2% of subjects, while the older women aged 50–65 years formed the rest of the sample. Most of the participants (77.7%) live in houses that afford them with an easy access to sunshine, while the remaining subjects live in apartments which are characterized by limited sunshine access. Monthly family income was 1000 USD or less for about half of the study subjects (48.8%) and more than 1000 USD for the rest of them. Regarding education, about one-fifth of participants (19.9%) had a college degree. However, education level did not exceed high school for 80.1% of the sample. In addition, about two-thirds of the participants (65.1%) were married, while unmarried women; including single, divorced and widowed women, composed 34.9% of the sample. Actually, 40.9% of the participating women were rarely or never exposed to the sunlight. Only 15.1% of them had frequent sun exposure. The morning was the usual daytime of sun exposure for about half of the study sample (50.3%) and the duration of sun exposure for 72.8% of the participants was less than 15 minutes per day. Finally, most of the subjects (72.9%) had no daily practice of exercise.

**Table 1:** Socio-demographic and lifestyle characteristics of study subjects (n = 166).

Variables	Study Subjects* N (%)
**Nationality**	
Saudi	142 (85.5%)
Non Saudi	24 (14.5%)
**Age Groups**	
30–49 years	95 (57.2%)
50–65 years	71 (42.8%)
**Type of Housing**	
House	129 (77.7%)
Apartment	37 (22.3%)
**Family Income**	
1000 USD or less	81 (48.8%)
More than 1000 USD	85 (51.2%)
**Education Level**	
High school education or less	133 (80.1%)
College education or more	33 (19.9%)
**Marital Status**	
Married	108 (65.1%)
Unmarried	58 (34.9%)
**Sun Exposure Frequency**	
Frequent	25 (15.1%)
Occasional	73 (44.0%)
Rare	49 (29.5%)
Never	19 (11.4%)
Daytime of Sun Exposure**	
Morning	74 (50.3%)
Midday	40 (27.2%)
Evening	33 (22.4%)
Daily Sun Exposure Duration**	
Less than 15 minutes	107 (72.8%)
15 minutes or more	40 (27.2%)
**Daily Exercise Practice**	
No	121 (72.9%)
Yes	45 (27.1%)

*Categorical variables were expressed as numbers and percentages.

**Women who never exposed to the sun were excluded (n = 19).

Health status characteristics and dietary supplements intake of the study subjects are shown in Table 2. Obesity was reported in 62.0% of the study sample. In addition, 24.1% of the participants had been diagnosed with type 2 diabetes, while hypertension was diagnosed among 15.7% of subjects. Vitamin D supplement was taken by 29.5% of the participants as a single nutrient supplement (at least 400 IU/day). Moreover, 18.1% of the subjects were taking the multi-vitamins supplement which contains variable amounts of vitamin D (at least 400 IU/day). The other supplementation that reported by subjects were calcium supplement (21.1%), and the multi-minerals supplement (7.8%) that provides at least 400 mg/day of calcium.

**Table 2:** Health status characteristics, and dietary supplement intake of study subjects (n = 166).

Variables	Study Subjects* N (%)
**Obesity (BMI ≥ 30)**	
No	63 (38.0%)
Yes	103 (62.0%)
**Type 2 Diabetes**	
No	126 (75.9%)
Yes	40 (24.1%)
**Hypertention**	
No	140 (84.3%)
Yes	26 (15.7%)
**Vitamin D Supplement**	
No	117 (70.5%)
Yes	49 (29.5%)
**Multi-vitamins Supplement**	
No	136 (81.9%)
Yes	30 (18.1%)
**Calcium Supplement**	
No	131 (78.9%)
Yes	35 (21.1%)
**Multi-minerals Supplement**	
No	153 (92.2%)
Yes	13 (7.8%)

*Categorical variables were expressed as numbers and percentages.

**Figure 1:**
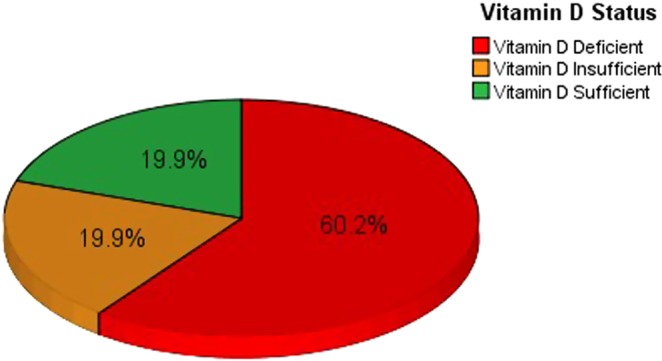
Pie chart illustrated vitamin D status among study subjects (n = 166).

Vitamin D deficiency (25(OH)D < 20 ng/mL) was reported in 60.2% of the participants; about half of them (28.9% of study sample) had severe vitamin D deficiency (25(OH)D < 10 ng/mL). Furthermore, 19.9% of subjects were found with vitamin D insufficiency (25(OH)D = 20–29.9 ng/mL) and another 19.9% of subjects were found with vitamin D sufficiency (25(OH)D ≥ 30 ng/mL) (Fig. 1). The mean of serum 25(OH)D concentrations of all subjects was 20.7 ng/mL (SD = 13.2), whereas the median was 16.9 ng/mL (IQR = 17.2) (Table 3). The only variable from the selected socio- demographic characteristics that significantly associated with vitamin D deficiency was age groups (Table 4). Younger women aged 30–49 years had a significantly higher risk of vitamin D deficiency than older women aged 50–65 years (odds ratio [OR] = 2.01, p = 0.03). Furthermore, one of selected lifestyle variables was associated with a higher risk of vitamin D deficiency but not significantly. Participants with a frequent sun exposure had a lower risk of vitamin D deficiency compared with those never exposed to sunlight (OR = 2.00). In fact, all variables related to health status characteristics and used in this study were not significantly associated with the risk of vitamin D deficiency. On the other hand, a lower risk of vitamin D deficiency was significantly associated with taking the supplements of vitamin D (OR = 3.14, p value = 0.001), multi-vitamins (OR = 2.32, p value = 0.04), or calcium (OR = 4.62, p value = 0.001) by the study subjects (Table 5).

**Table 3:** Statistical measures of serum 25(OH)D for total study subjects and the three subgroups of vitamin D status: sufficient; insufficient, and deficient*.

Statistical Measures of Serum 25(OH)D (ng/mL)	Total Study Subjects (n = 166)	Vitamin D Sufficient (n = 33)	Vitamin D Insufficient (n = 33)	Vitamin D Deficient (n = 100)
Mean ± SD	20.7 ± 13.2	42.9 ± 8.2	24.9 ± 2.9	11.9 ± 4.4
Median ± IQR	16.9 ± 17.2	41.3 ± 10.2	25.0 ± 5.3	11.5 ± 7.7

*Values can be converted to SI units (nmol/L) by multiplying by 2.5.

**Table 4:** Risk of vitamin D deficiency among study subjects for socio-demographic and lifestyle variables.

Variables	Odds Ratio (95% CI)	*p* value*	95% CI for Odds Ratio	
			Lower	Upper
**Nationality**				
Saudi	1.34	0.51	0.56	3.20
Non Saudi	1			
**Age Groups**				
30–49 years	2.01	**0.03**	1.07	3.78
50–65 years	1			
**Type of Housing**				
House	1	0.52		
Apartment	1.29		0.60	2.76
**Family Income**				
1000 USD or less	1.13	0.7	0.61	2.10
More than 1000 USD	1			
**Education Level**				
High school education or less	1	0.96		
College education or more	1.02		0.47	2.22
**Marital Status**				
Married	1	0.72		
Unmarried	1.13		0.59	2.17
**Sun Exposure Frequency**				
Frequent	1	0.69		
Occasional	1.32		0.53	3.30
Rare	1.59		0.60	4.22
Never	2.00		0.58	6.95
**Daytime of Sun Exposure**				
Morning	1	0.32		
Midday	1.28		0.58	2.78
Evening	1.96		0.82	4.68
**Daily Sun Exposure Duration**				
Less than 15 minutes	1.27	0.53	0.61	2.64
15 minutes or more	1			
**Daily Exercise Practice**				
No	1	0.75		
Yes	1.12		0.56	2.27

*Differences were considered statistically significant at *p* value < 0.05 and significants values were presented in **Bold** type.

**Table 5:** Risk of vitamin D deficiency among study subjects for health status variables and dietary supplement intake.

Variables	Odds Ratio (95% CI)	*p* value*	95% CI for Odds Ratio	
			Lower	Upper
**Obesity (BMI ≥ 30)**				
No	1.25	0.5	0.65	2.38
Yes	1			
**Type 2 Diabetes**				
No	1	0.74		
Yes	1.13		0.55	2.36
**Hypertention**				
No	1	0.31		
Yes	1.77		0.74	2.58
**Vitamin D Supplement**				
No	3.14	**0**.**001**	1.57	6.25
Yes	1			
**Multi-vitamins Supplement**				
No	2.32	**0**.**04**	1.04	5.18
Yes	1			
**Calcium Supplement**				
No	4.62	**0**.**001**	2.07	10.32
Yes	1			
**Multi-minerals Supplement**				
No	1.68	0.29	0.60	5.80
Yes	1			

*Differences were considered statistically significant at *p* value < 0.05 and significants values were presented in **Bold** type.

## Discussion

The present study showed a high prevalence of vitamin D deficiency among women living in Riyadh, Saudi Arabia. Actually, 60.2% of women who participated in this study had hypovitaminosis D. Our result is consistent with a recent systematic review that carried out to determine the prevalence of hypovitaminosis D in the Saudi population based on the currently available literature which suggest that the vitamin D deficiency is around 60%^15^. Vitamin D deficiency should not be widespread in Saudi Arabia as a country with ample sunshine that is available most days of the year^11^. However, accumulating evidence shows that hypovitaminosis D is a serious public health problem in the population of Saudi Arabia^16–19^. Historically, the early report published in 1984 indicated a possible hypovitaminosis D incidence among women in Saudi Arabia^20^. After more than three decades, the prevalence of vitamin D deficiency in Saudi Arabia is still unresolved and might have even increased^11,12,15,21^. Only a few studies had used population-based samples to assess hypovitaminosis D among women in Saudi Arabia^22,23^. In a survey (n = 1172) targeted healthy Saudi women during June 2008 to June 2009 in the city of Jeddah, 80% of subjects had a hypovitaminosis D (25(OH)D < 20 ng/mL)^22^. Another study was conducted in 2009 to evaluate vitamin D status among a large sample (n = 1556) of Saudi women living in Riyadh. They reported high rates of vitamin D deficiency (25(OH)D < 20 ng/mL) among women during both the summer (68–80%) and the winter (76–85%) seasons^23^.

Because there are limited studies that show prevalence of vitamin D deficiency in Saudi Arabian women^12^, the present study provides an important contribution in this direction. An interesting finding in this study was that younger women (30–49 years) had a significantly higher risk of hypovitaminosis D than older women (50–65 years). The same trend was reported previously among the population in Saudi Arabia^16,19,22–24^. This finding might be related to more vitamin D supplement taken by older women^23^. Moreover, young women in Saudi Arabia usually have a relatively low dietary intake of vitamin D^25^. This low dietary intake of vitamin D could be related to their frequent consumption of energy-dense foods such as fast foods and soft drinks which are poor in vitamin D^26,27^, while they have low consumption of healthy foods that contain reasonable amounts of vitamin D such as dairy products which are mostly fortified with vitamin D in Saudi Arabia^27,28^. Fatty Fish such as salmon and mackerel has been known as a rich source of vitamin D^29^. Saudi dietary pattern is generally characterized by lack of fish consumption especially in landlocked areas such as Riyadh city. Although the association was not significant, our results revealed that women who were never exposed to sunlight were at higher risk of hypovitaminosis D compared with those exposed to sunlight frequently (OR = 2.00). Sun exposure avoidance is a common lifestyle practice in Saudi Arabia especially among women due to many reasons including; modern lifestyles which focus mainly on indoor sedentary activities, hot climate which limit outdoor activities during the daytimes, aesthetic reasons where women commonly use sun blockers as they prefer fair skin rather than suntanned skin, and cultural reasons related to dress style of women which tend to be usually dark and covered the entire body^23,30–33^.

As expected, taking supplements of either vitamin D or multi-vitamins that contain vitamin D were led to a significantly lower risk of hypovitaminosis D. Unfortunately, it is difficult for most people to achieve recommended levels of vitamin D intake, even when they consume a healthy and balanced diet because rich dietary sources of vitamin D are not common^3^. Therefore, food fortification and dietary supplementation of vitamin D are considered as acceptable strategies to enhance vitamin D status among the general population^34^. Currently, food fortification with vitamin D is not mandatory and most foods sold at Saudi markets which claim to be fortified with vitamin D are either not fortified at all or contain small amounts of vitamin D compared to guidelines set for the United States markets^35^. In addition, vitamin D supplement is generally not common^36^. Furthermore, there are no recommendations regarding vitamin D supplementation issued by the public health care system consequently there is a variation in the recommendations about vitamin D supplementation in different health-care institutions^19,37^. Hence, taking vitamin D supplement under medical supervision by women at high risk of hypovitaminosis D should be considered in order to combat the high prevalence of vitamin D deficiency^38,39^. A recent study revealed that vitamin D supplementation was the most effective strategy to improve vitamin D status in Saudi adults and children compared to sunlight exposure or vitamin D fortified dairy products consumption^40^. Remarkably, taking calcium supplement was significantly associated with a lower risk of hypovitaminosis D among participants. The same finding was reported elsewhere^27^. This finding could be referring to a common practice in Saudi Arabia where many clinicians usually prescribed vitamin D supplement along with calcium supplement for women to protect them against osteoporosis, without conducting any laboratory assessment for vitamin D status^23^. Our data support this explanation as 46.9% and 43.3% of subjects who consumed calcium supplement were concurrently taking vitamin D supplement and multi-vitamins supplement respectively (data were not presented in results section nor tables).

In light of a high prevalence of vitamin D deficiency in Saudi Arabia, there is an urgent need for a national strategy to prevent and manage this serious health problem. One important target for this strategy must be working to raise the public awareness about vitamin D^12^. Lack of awareness among health-care providers and the general population regarding vitamin D contributes to raising the prevalence of vitamin D deficiency in Saudi Arabia^33,37^. All efforts must be dedicated to educating the community on how to prevent hypovitaminosis D through adapting proper lifestyle practices focused on maintaining an adequate sun exposure and increasing their dietary intake of vitamin D form through dietary sources such as seafood and vitamin D fortified foods like dairy products^36^. Other targets for this national strategy could be creating a national approach related to vitamin D fortification and supplementation. Governmental regulations are required to determine acceptable levels of food fortification with vitamin D and it is important to monitor the enforcement for these regulations by the food manufacturers and importers in Saudi Arabia. Furthermore, there is a necessity to generate evidence-based national recommendations of vitamin D supplementation and applying them in all health-care institutions in the country. Indeed, the public health care system in Saudi Arabia should seriously consider screening the status of vitamin D among women at high risk and treating those have a deficiency by prescribing them a suitable supplementation of vitamin D^39^. Thus, developing a relevant screening tool that can be used in daily clinical practice to detect individuals at high risk of hypovitaminosis D should be highly recommended^41^.

This study had a few limitations. The first one is the small sample size. Second, the vitamin D assay was done using the radioimmunoassay technique, which may lead to a considerable persistent proportional bias in the results^42^. However, this study still provides valuable data about risk factors might be associated with vitamin D deficiency among women living in Riyadh, Saudi Arabia.

In conclusion, the prevalence of vitamin D deficiency among women living in Riyadh, Saudi Arabia was high. Older age and taking the supplements of vitamin D, multi-vitamins or calcium were identified as factors that linked with a lower risk of hypovitaminosis D among women living in Riyadh, Saudi Arabia. A national strategy is needed to control a hypovitaminosis D crisis in a country with plentiful sunshine throughout the year. It is important to raise public awareness among women about rich dietary sources of vitamin D, and the sun exposure recommendations. Furthermore, governmental efforts to regulate and enhance of vitamin D fortification and supplementation across the country would also be useful to prevent and manage this serious health problem. Finally, the public health care system in Saudi Arabia should play an active role in screening vitamin D status among women at high risk of hypovitaminosis D by treating those have a deficiency by prescribing a suitable dose of vitamin D supplement.

## Data Availability

Please contact the corresponding author for data requests.
